# A Simple, Affordable, Rapid, Stabilized, Colorimetric, Versatile RT-LAMP Assay to Detect SARS-CoV-2

**DOI:** 10.3390/diagnostics11030438

**Published:** 2021-03-04

**Authors:** Juan García-Bernalt Diego, Pedro Fernández-Soto, Marta Domínguez-Gil, Moncef Belhassen-García, Juan Luis Muñoz Bellido, Antonio Muro

**Affiliations:** 1 Infectious and Tropical Diseases Research Group (e-INTRO), Biomedical Research Institute of Salamanca-Research Centre for Tropical Diseases at the University of Salamanca (IBSAL-CIETUS), Faculty of Pharmacy, University of Salamanca, 37007 Salamanca, Spain; juanbernalt95@usal.es (J.G.-B.D.); belhassen@usal.es (M.B.-G.); ama@usal.es (A.M.); 2 Microbiology Service, Hospital Universitario Río Hortega, 47012 Valladolid, Spain; mdominguezgilgo@saludcastillayleon.es; 3 Internal Medicine Service, Sección de Enfermedades Infecciosas, Complejo Asistencial Universitario de Salamanca, University of Salamanca, 37007 Salamanca, Spain; 4 Microbiology and Parasitology Service, Complejo Asistencial Universitario de Salamanca, University of Salamanca, 37007 Salamanca, Spain; jlmubel@usal.es

**Keywords:** SARS-CoV-2, RT-LAMP, molecular diagnostics, dry-RT-LAMP, point-of-care

## Abstract

The SARS-CoV-2 pandemic has forced all countries worldwide to rapidly develop and implement widespread testing to control and manage the Coronavirus Disease 2019 (COVID-19). reverse-transcription (RT)-qPCR is the gold standard molecular diagnostic method for COVID-19, mostly in automated testing platforms. These systems are accurate and effective, but also costly, time-consuming, high-technological, infrastructure-dependent, and currently suffer from commercial reagent supply shortages. The reverse-transcription loop-mediated isothermal amplification (RT-LAMP) can be used as an alternative testing method. Here, we present a novel versatile (real-time and colorimetric) RT-LAMP for the simple (one-step), affordable (~1.7 €/sample), and rapid detection of SARS-CoV-2 targeting both *ORF1ab* and *N* genes of the novel virus genome. We demonstrate the assay on RT-qPCR-positive clinical samples, obtaining most positive results under 25 min. In addition, a novel 30-min one-step drying protocol has been developed to stabilize the RT-LAMP reaction mixtures, allowing them to be stored at room temperature functionally for up to two months, as predicted by the *Q*_10_. This Dry-RT-LAMP methodology is suitable for potentially ready-to-use COVID-19 diagnosis. After further testing and validation, it could be easily applied both in developed and in low-income countries yielding rapid and reliable results.

## 1. Introduction

Coronavirus disease 19 (COVID-19) is an infection caused by the novel coronavirus SARS-CoV-2, which emerged in China in December of 2019, becoming the seventh member of the *Coronaviridae* family to infect humans [1]. Although it is less severe than other previously described coronaviruses infecting humans, such as SARS or MERS coronaviruses, it has a significantly higher transmission capacity. This elevated transmission compelled the World Health Organization (WHO) to declare a global health emergency on 31 January 2020 and, subsequently, a pandemic situation on 11 March 2020 [2]. Until the date of writing this manuscript, the COVID-19 pandemic had already affected over 114 million people worldwide and caused over 2.5 million deaths (https://coronavirus.jhu.edu/map.html; accessed on 2 March 2021).

For COVID-19, a mean incubation period of 6.4 days (ranging from 2.1 to 11.1 days) is estimated [3]. Clinical manifestations can range from mild flu-like symptoms to severe or critical, and patients can present an either symptomatic or asymptomatic infection. The later seem to account for 40 to 45% of cases [4]. The most prevalent symptoms are fever, cough (either productive or not) and myalgia or fatigue, but other signs such as headache, hemoptysis and diarrhea may appear [5,6]. In severe presentations, COVID-19 is associated with pneumonia and acute respiratory distress syndrome (ARDS), for which elderly and chronic disease patients are particularly susceptible [7]. SARS-CoV-2 can also affect the gastrointestinal, nervous, or cardiovascular systems [6]. Timely diagnosis in the early infection stages is hindered by the aforementioned incubation period together with the asymptomatic course or unspecific manifestations of the illness in a high proportion of patients [5]. Early diagnosis allows a prompt intervention, reducing the risk of developing more serious complications. Moreover, one of the main challenges to contain the spread of COVID-19 is the identification of asymptomatic cases.

In that sense, nucleic acid detection-based tests are reliable and accurate approaches for viral infection detection. Specifically, reverse-transcription (RT)-qPCR is the main molecular method used for the detection of all kinds of coronaviruses, including SARS-CoV-2 [8,9]. Currently, COVID-19 RT-qPCR-based tests target mainly the ORF1ab region of SARS-CoV-2 genome, combined with genes coding for the E and N proteins. However, protocols for commercial RT-qPCR kits uses different reagents and different combination of the aforementioned genes depending on the country [10]. Moreover, the devices to perform RT-qPCR result in a wide range of costs and processing times, as well as variations in tests accuracy [11]. Moreover, RT-qPCR technology is not easily adaptable for point-of-care diagnosis in low-resource settings due to the need for temperature cycling. An increasingly recognized alternative is loop-mediated isothermal amplification (LAMP) technology, a highly efficient, specific, and rapid technology to amplify DNA at a constant temperature, using two or three primer sets and a *Bst* polymerase with high strand displacement activity [12]. Many prominent advantages of LAMP over PCR-based technologies in terms of sensitivity, specificity, rapidity, robustness, and cost have been extensively informed [13]. Additionally, different approaches have been developed to allow LAMP reagents storage, in a single tube at room temperature over extended periods, to be used for point-of-care testing [14]. Reverse-transcription LAMP (RT-LAMP) combines LAMP to amplify DNA from an RNA target in one-step reaction by directly adding a dedicated reverse transciptase^10^ or a DNA polymerase with reverse transcriptase activity to the reaction mixture. RT-LAMP shares the versatility and all the benefits of LAMP technology and has already been developed for the detection of numerous RNA viruses including virus influenza, Zika, Ebola, and MERS [15]. The prominent prospect of RT-LAMP in the context of COVID-19 diagnosis has been recently discussed by Augustine et al. [16].

Thus, since the outbreak of COVID-19, in parallel with the emergence of new in-house and commercial RT-qPCR assays to detect SARS-CoV-2 RNA, numerous RT-LAMP assays have been rapidly developed mainly targeting ORF1ab [17,18,19,20,21,22,23,24] and gene N [17,19,22,25,26,27,28,29,30] sequences, which are the regions recommended for RT-qPCR by the Centre of Disease Control and Prevention (CDC, Atlanta, USA) [31]. Although less explored, some assays have also targeted Gen S [17,19], Gen E [30] and Gen M sequences [32]. A comparison of different RT-LAMP for SARS-CoV-2 detection, including master mixes, primer sets, targeting genes, readout monitoring, and analytical sensitivity has been recently summarized and examined by Thompson & Lei [33]. Those studies expose a wide variety of methodologies, sensitivities, and results. Furthermore, a commercial technology based on RT-LAMP, ID NOW COVID-19 (ABBOTT LABORATORIES, Chicago, USA) has been granted the Emergency Use Authorization (EUA) by the FDA [9]. RT-LAMP in combination with other molecular techniques such as CRISPR-Cas12 [30] or recombinase polymerase assay (RPA) [24], has also been optimized for SARS-CoV-2 detection. Additionally, various strategies to avoid RNA extraction and purification, one of the main bottlenecks molecular testing is facing now, have been presented, mainly for nasopharyngeal swabs [24] and saliva [34] analyses.

In this paper, with the aim of contributing to an effective COVID-19 diagnosis, we present a novel, specific, sensitive, rapid, and versatile RT-LAMP assay for SARS-CoV-2 RNA detection. We demonstrate our RT-LAMP assay proper operation on clinical samples by using a portable real-time device and in conventional colorimetric trials easily visualized with the naked eye. Furthermore, we developed a new simple one-step desiccation procedure to stabilize the RT-LAMP reagents in a single tube for potentially ready-to-use COVID-19 diagnosis. The Dry-RT-LAMP methodology does not require complex instrumentation and it is much faster to perform (30 min) than other available alternatives such as lyophilization. The Dry-RT-LAMP format could be very useful for easy testing in situations of high diagnostic demand and in low-resources settings thus contributing to rapid diagnosis for COVID-19.

## 2. Materials and Methods

### 2.1. Clinical Specimens

Nasopharyngeal swabs specimens were collected in Sample Preservation Solution (MOLE BIOSCIENCE, SUNGO Europe B.V., Amsterdam, The Netherlands) as part of the routine testing of patients for COVID-19 at the University Hospital of Salamanca, Salamanca, Spain. Collected samples were delivered to the Laboratory of Microbiology, and then processed in a biosafety level 2 cabin until inactivation by mixing with a lysis buffer.

### 2.2. RNA Isolation and RT-qPCR Amplification

Samples were processed either by performing RNA isolation (NUCLISENS EASYMAG, BIOMÉRIEUX, France) and RT and amplification (VIASURE SARS-CoV-2 Real-Time PCR Detection Kit, CERTEST BIOTECH, Spain) separately, or in an integrated way in an automated platform (COBAS 6800, ROCHE, Switzerland) following manufactures’ instructions. RT-qPCR for the detection of SARS-CoV-2 RNA was performed using commercial kits and reagents targeting ORF1ab and gen N (VIASURE SARS-CoV-2) or ORF1ab and gen E (COBAS SARS-CoV-2 Test) following manufacturers’ instructions. Aliquots of purified RNA samples were stored at −80 °C until further analysis. For RNA samples analyzed, SARS-CoV-2 Cycle threshold (Ct) values for ORF1ab, E, or N amplified targets were used as reference for the RT-LAMP assays.

### 2.3. Viral RNA-Positive Control and Patients’ RNA Samples Selected

An RNA isolate amplified by VIASURE SARS-CoV-2 Real-Time PCR Detection Kit from a positive COVID-19 patient with a Ct = 25 min for ORF1ab target and a Ct = 29 min for gen N was selected as a well-established RNA-positive control to set up the COVID-LAMP assays (hereafter, C+). The Ct values allowed us to determinate a concentration between 10^5^ and 10^4^ copies per RT-qPCR reaction (cpr) (5 μL of sample), attending to the VIASURE SARS-CoV-2 Real-Time PCR Detection Kit’s handbook (https://www.certest.es/wp-content/uploads/2020/03/IU-NCO212enes0420-rev.01.pdf, accessed on 10 November 2020). Therefore, a concentration of 2 × 10^4^ cpr (1 μL of C+) was estimated for analytical sensitivity calculations.

To further test COVID-LAMP effectiveness, 20 RNA isolates from COVID-19 patients were selected and distributed into four groups according to Ct values obtained from RT-qPCR: group 1 (*n* = 6), RT-qPCR-positive for both ORF1ab (Ct ≤ 30 min) and E/N genes; group 2 (*n* = 6), RT-qPCR-positive for both ORF1ab (Ct > 30 min) and E/N genes; group 3 (*n* = 2), RT-qPCR-positive for ORF1ab and RT-qPCR-negative for gen E; group 4 (*n* = 6), RT-qPCR-negative for ORF1ab and RT-qPCR-positive for N gene. Groups of samples, Ct values for amplified targets, and commercial kits used in RT-qPCR are indicated in Table 2.

### 2.4. RT-LAMP Primer Design

**Figure 1:**
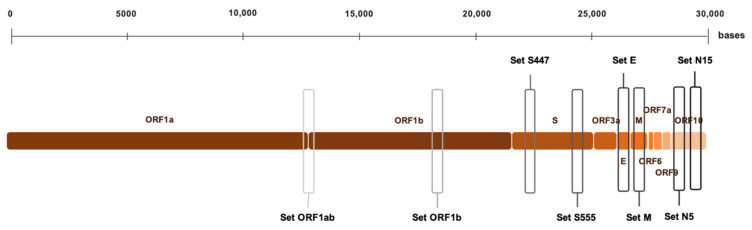
Schematic representation of COVID-LAMP target localization within SARS-CoV-2 genome. Genbank sequence accession number: MN908947.3 [35].

Primer sets used for LAMP were based on the SARS-CoV-2 complete genome sequence from the NCBI nucleotide database (GenBank: MN908947.3) [35] to target specific regions in ORF1ab, ORF1b, S, E, M, and N genes. The primer set used to target the conserved sequence of ORF1ab was previously described by El-Tholoth et al. [24]. Primer sets targeting ORF1b and S genes were original designs, based upon recently reported sequences used for RT-qPCR detection of SARS-CoV-2 by Wu et al. [35], with the appropriate modifications to fit RT-LAMP constrains. Primer sets targeting E, M, and N genes were also original designs. All primer sets were designed using the open access Primer Explorer V5 software tool (EIKEN CHEMICAL Co., Ltd., Tokyo, Japan) at the website: https://primerexplorer.jp/e/; accessed on 1 May 2020. Once the best parameters have been considered, single primer sets were selected for ORF1ab, ORF1b, E, and M genes; for S (S447, S555) and N (N5, N15) genes two primer sets targeting different regions within each gene were selected. Each primer set included two outer primers (F3 and B3), two inner primers (FIP and BIP) and, for ORF1ab, S447, N5, and N15 primer sets, two additional loop primers (LF and LB) were designed and selected. All the primers were synthesized (synthesis scale, 0.025 µmol; purification, desalt; solution, water) by EUROFINS GENOMICS (Ebersber, Germany). The localization of LAMP targets on the genome of SARS-CoV-2 is represented in Figure 1. Sequences of the oligonucleotide primer sets finally selected are listed in Table 1.

### 2.5. RT-LAMP Reaction

For the one-step RT-LAMP reaction, two reaction mixtures containing different polymerases were evaluated. On one hand, RT-LAMP assay was performed using *Bst* 3.0 DNA Polymerase (*Bst* 3.0) (NEW ENGLAND BIOLABS Ltd., Ipswich, USA) for both isothermal amplification performance and reverse-transcription. On the other hand, RT-LAMP assays were performed in the presence of two enzymes: *Bst* 2.0 WarmStart DNA Polymerase (*Bst* 2.0 WS) and WarmStart *RTx* Reverse Transcriptase (*RTx* WS) (NEW ENGLAND BIOLABS Ltd., Ipswich, USA). Briefly, RT-LAMP reaction mixtures (15 μL) contained 1.6 μM FIP/BIP primers, 0.2 μM F3/B3 primers, 0.4 μM LF/LB primers (if applicable), 1.4 mM of each dNTP, (BIORON GmBH, Römerberg, Germany) 0.13 M of D-(+)-Trehalose dihydrate (Sigma-Aldrich, USA) (from now on, trehalose), 6mM MgSO_4_, and 1× Isothermal Amplification Buffer II (20 mM Tris-HCl (pH 8.8), 150 mM KCl, 10 mM (NH4)_2_SO_4_, 2 mM MgSO_4_, 0.1% Tween20) for *Bst* 3.0 DNA polymerase (0.32 U/μL) or 1× Amplification Buffer (20 mM Tris-HCl (pH 8.8), 50 mM KCl, 10 mM (NH4)_2_SO_4_, 2 mM MgSO_4_, 0.1% Tween20) for *Bst* 2.0 WS (0.32 U/μL) and *RTx* WS (0.3 μL), with 1 μL of template RNA (C+, for positive control; ultrapure water for negative control). To establish the optimal reaction conditions, the one-step RT-LAMP assay was real-time evaluated at different temperatures and reaction times in 8-tube Genie Strips on a portable Genie III device (OPTIGENE Ltd., Horsham, UK). In this case, 0.24 μL of EvaGreen 20× in water (BIOTIUM, San Francisco, USA) was added to the reaction mix before the reaction started. For *Bst* 3.0 reactions, temperatures ranging 62 °C to 72 °C (in 2 °C increments) and reaction times of 45, 60, and 80 min were tested. For *Bst* 2.0 WS and *RTx* WS reactions, different temperatures (63 °C, 64 °C, 65 °C) and reaction times (45, 60, 80 min) were also tested. All reactions were performed in duplicates.

In addition, conventional colorimetric one-step RT-LAMP reactions were also performed with *Bst* 2.0 WS and *RTx* WS in a heating block. For this, attending to samples availability limitations, eight RNA isolates representing the four groups of COVID-19 patients’ samples used in the study were selected. Results were visually inspected by the naked eye based on the color change observed (green for a positive result and orange for a negative result) with 1 μL of SYBR Green I 1000× (INVITROGEN, USA) added post-amplification to each tube. To avoid potential cross-contamination with amplified products, the tubes were briefly centrifuged and carefully opened in a laminar flow hood before adding the dye.

### 2.6. Sensitivity and Specificity

To assess the analytical sensitivity of the primer sets in the detection of SARS-CoV-2, serial dilutions (10-fold) of the C+ in nuclease-free water (diluted from 1× to 10^−4^) were prepared and used to determine the limit of detection of the RT-LAMP assays.

To confirm the specificity of the evaluated primers a BLASTN local search and alignment analysis was carried out in GenBank online databases (NCBI; http://blast.ncbi.nlm.nih.gov/Blast.cgi; accessed on 1 September 2020) against currently available nucleotide sequences for other human respiratory viruses and other human-infecting viruses. Comparison included: Influenza A virus (taxid:11320), Influenza B virus (taxid:11520), Human parainfluenza virus 1 strain Washington/1964 (taxid:188538), Human parainfluenza virus 2 (strain Toshiba) (taxid:11214), Human parainfluenza virus 4a (taxid:11224), Human adenovirus 4a (taxid:35263), Human adenovirus 7 (taxid:10519), Enterovirus A (taxid:138948), Enterovirus B (taxid:138949), Enterovirus C (taxid:138950), Metapneumovirus (taxid:162387), Respiratory Syncytial Virus (taxid:12331), Zika virus (taxid:64320), Dengue virus (taxid:12637), Chikungunya virus (taxid:37124), and Middle East respiratory syndrome-related coronavirus (MERS-CoV) (taxid:1335626).

Additionally, the RT-LAMP primer sets were cross-tested for specificity against a panel of 13 RNA isolates of related coronaviruses obtained from patients infected with Coronavirus NL63, Coronavirus OC43, Bocavirus, Rinovirus, Metapneumovirus, Respiratory Syncytial Virus A, Respiratory Syncytial Virus B, Enterovirus, Parainfluenzae 1, Influenza H1N1, Influenza A H3, Influenza A H1, and Influenza B. These RNA isolates were provided by the laboratory of the National Influenza Centre of Valladolid (University Clinical Hospital of Valladolid, Valladolid, Castilla y León, Spain). This laboratory is part of a network of 126 laboratories around the world linked to the WHO responsible for the characterization and diagnosis of circulating influenza viruses.

### 2.7. Stabilization for Long-Term Room-Temperature Storage: Dry-RT-LAMP

We optimized the RT-LAMP reaction protocol for potential ready-to-use COVID-19 RT-LAMP test. For long-term room temperature storage, the master mixes containing the two enzymes and primer sets ORF1ab, N5, or N15 were stabilized by a vacuum process without centrifugation (so called, desiccation) in a Concentrator Plus (EPPENDORF, Hamburg, Germany) at RT for 30 min, following a single dry-up step as previously described elsewhere [14], with some modifications. In brief, RT-LAMP master mixes were dried in open 8-tube Genie Strips (OPTIGENE, Horsham, UK) separately in two partial mixes: one containing primers, dNTPs, and polymerases placed in the bottom of the tube in the presence of 1.8 μL of trehalose 2M; other containing Isothermal Buffer 10×, MgSO_4_, and 0.24 μL EvaGreen 10× in the tube cap in the presence of 2.25 μL of trehalose 2M. The desiccation procedure yielded two stable and well-adhered pellets in both cap and bottom of the tubes. To estimate the stability and functionality over time, the desiccated 8-tube strips were stored at 25 °C, 37 °C, and 45 °C for up to 28 days in paperboard storage boxes with some Silica Gel desiccant pouches inside to protect against moisture until use. After rehydration with ultrapure water (for negative controls) or ultrapure water containing RNA (for C+ or RNA samples), the real-time RT-LAMP assays with Genie III were performed at 63 °C for up to 120 min at 0, 1, 7, 14, 21, and 28 days post-desiccation.

### 2.8. Estimation of the Shelf-Life of the Dry-RT-LAMP Mixes

To estimate the shelf-life of the dry-reagent RT-LAMP mixes the accelerated ageing technique (also known as *Q*_10_ method) described by Clark (1991) was employed [36]. The shelf-life can be determined by either a real-time or an accelerated ageing test where Arrhenius Law is applied in a simulated environment. The method was conducted by exposing the dry-reagent RT-LAMP mixes to different temperatures (25 °C, 37 °C, 45 °C) for up to 28 days and assessing the functionality of the dried RT-LAMP reagents periodically, at 0, 1, 7, 14, 21, and 28 days post-desiccation. Data obtained were used to calculate the assay stability at RT (25 °C) with the following formulas:

$$AF=Q_{10}^{\left[0.1\times \left(Te-Ta\right)\right]}$$

$$AG=t_{e}\times AF$$

$$Estimated\;Shelf-life=AG+t_{e}$$

where *AF* is the acceleration factor used to correlate the shelf-life of the product at a lower temperature than the one used to perform the experiment; *Q*_10_ factor measures the temperature sensitivity of an enzymatic reaction rate due to an increase by 10 °C; *T_e_* represents the elevated temperature; *AG* is the accelerated age; *t_e_* is the length of time storage at elevated temperature, and *T_a_* is the ambient temperature (RT; 25 °C). Since for most biological reactions *Q*_10_ ~ 2 or 3 [37], we established a conservative value for *Q*_10_ = 1.9 to perform all the calculations. The evaluation of the estimated shelf-life was performed for dried master mixes mentioned above for up to 28 days using C+ as template. Additionally, dried N15-RT-LAMP master mixes for up to one week were also tested using RNA isolates as templates.

## 3. Results

### 3.1. RT-LAMP Primer Sets Screening and Selection

The first screening of each primer set designed was performed using the *Bst* 3.0 DNA polymerase in real-time conditions to amplify C+ template at different temperatures and reaction times described in the ‘Methods’ section. Frequently, non-specific amplification was obtained, and results were rather irregular and not reproducible. Therefore, further use in RT-LAMP amplification of SARS-CoV-2 RNA was discarded.

**Figure 2:**
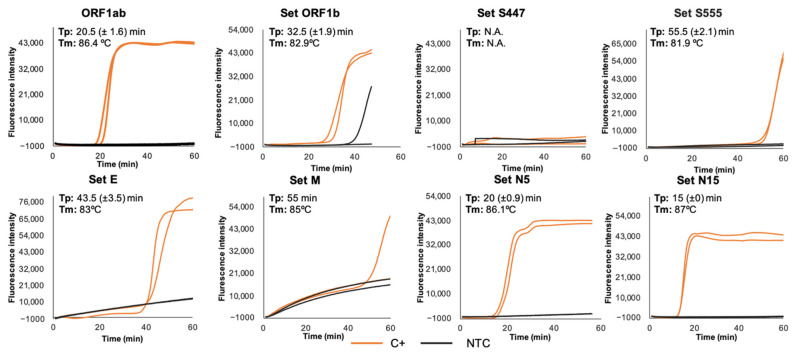
Real-time RT-LAMP assays performed using the eight primer sets evaluated for the detection of SARS-CoV-2. EvaGreen 20× fluorescence signal over time for primer sets ORF1ab, ORF1b, S447, S555, E, M, N5, and N15 is shown. Orange lines (C+, positive control); black lines (NTC, non-template control). Time to positivity with standard error (Tp (±SE); min) and melting temperatures (Tm; °C) for each primer set are indicated. All reactions were performed in duplicates.

The second screening of each primer set was also performed in real-time conditions using the combination of the *Bst* 2.0 WS and RT × WS. After testing different RT-LAMP conditions, a reaction time of 60 min at 63 °C plus 5–10 min of inactivation was considered the most appropriate for performance comparison among primer sets. Subsequently, the accuracy and efficiency of each primer set was evaluated through RT-LAMP using C+ as template in duplicate. Results obtained are shown in Figure 2. Four of the eight primer sets were selected for further evaluation based on the shorter time to positivity (Tp)—thus meaning the fastest amplification—reproducibility and the absence of non-specific amplifications: set ORF1ab (Tp = 20.5 min), set E (Tp = 43.5 min), set N5 (Tp = 20 min), and set N15 (Tp = 15 min). The remaining primer sets were discarded for further assessment because of relatively early non-specific amplifications (set ORF1b), failed amplification (set S447), poor amplification and reproducibility (set M), or long Tp value (set S555).

### 3.2. Sensitivity and Specificity of RT-LAMP

**Figure 3:**
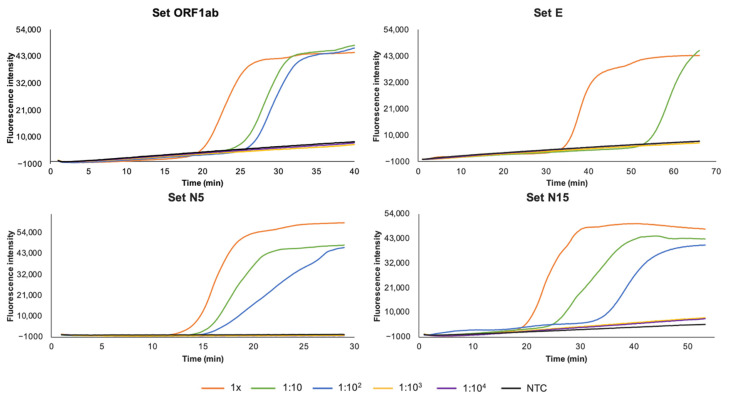
Sensitivity assessment of the RT-LAMP assays for SARS-CoV-2 RNA detection using primer sets ORF1ab, E, N5, and N15. The 10-fold dilutions (1×-1:10^4^) of positive control (C+) are represented by different color lines; non-template control (NTC) is represented by black lines.

Regarding to the sensitivity of the RT-LAMP assays, 10-fold serial dilutions of C+ was amplified by real-time RT-LAMP to determine the lower limit of detection. Analytical sensitivities for selected primer sets ORF1ab, E, N5, and N15 are shown in Figure 3. The results indicated that RT-LAMP assays using sets ORF1ab, N5, and N15 were 10 times more sensitive than RT-LAMP assay using set E (1:100 vs. 1:10 dilution, respectively). According to this, an approximate limit of detection of 2 × 10^2^ cpr for RT-LAMP using sets ORF1ab, N5, and N15, and 2 × 10^3^ cpr for RT-LAMP using set E was established.

**Figure 4:**
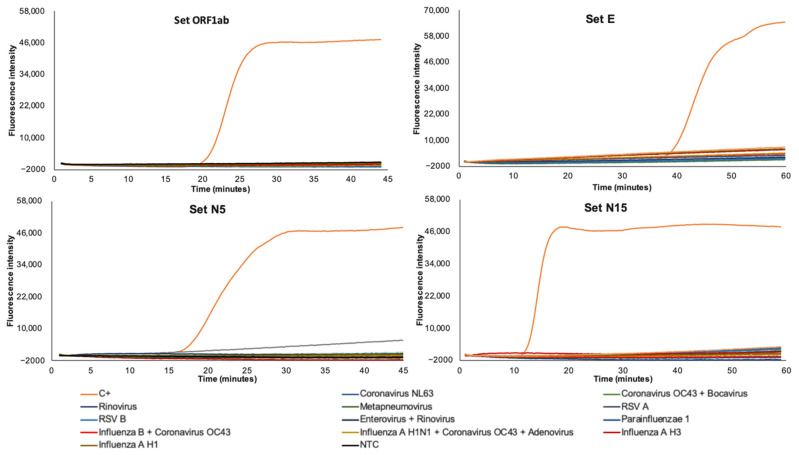
Specificity assessment of the RT-LAMP assays for SARS-CoV-2 RNA detection using primer sets ORF1ab, E, N5, and N15. A panel of 13 purified RNA isolates of related viruses obtained from infected patients are included: Coronavirus NL63, Coronavirus OC43, Bocavirus, Rinovirus, Metapneumovirus, Respiratory Syncytial Virus A, Respiratory Syncytial Virus B, Enterovirus, Parainfluenzae 1, Influenza H1N1, Influenza A H3, Influenza A H1, and Influenza B. One sample contained RNA from two viruses (Influenza B + Coronavirus OC43); other sample contained RNA from three viruses (Influenza A H1 + Coronavirus OC43 + Adenovirus).

No significant similarity between targets selected for SARS-CoV-2 detection and other sequences reported for possible human-infecting viruses was in silico detected, when searching in databases. Furthermore, no RNA isolates from patients infected with related coronaviruses was amplified when using the selected primer sets that resulted in the most efficient in amplifying SARS-CoV-2 RNA (set ORF1ab, set E, set N5, set N15), thus indicating the high specificity of the established RT-LAMP assays (Figure 4).

### 3.3. Clinical Samples Testing

Based on the analysis with RT-qPCR, 20 RNA isolates from COVID-19 patients were analyzed by RT-LAMP with the most efficient primer sets in this study: set ORF1ab, set E, set N5, and set N15. The comparison of the Ct values obtained by RT-qPCR and Tp values of RT-LAMP assays is shown in Table 2. When testing the six RT-qPCR-positive samples of group 1 (ORF1ab+; Ct ≤ 30/E+ or N+) the RT-LAMP assays using primer sets ORF1ab, N5, and N15, each detected 6/6 (100% sensitivity) with shorter Tp values for all samples than Ct values obtained by RT-qPCR. It needs to be highlighted that Tp for RT-LAMP includes both retro-transcription and amplification processes in a one-step reaction while Ct of RT-PCR accounts only for the amplification time but not retro-transcription time. The RT-LAMP using primer set E detected 5/6 samples (83.3% sensitivity), with a long Tp = 72 min for sample 2, the only one tested by COBAS RT-qPCR for gene E (Ct = 26) in this group. The Ct values for the four remaining positive samples for gene E resulted equal or higher than those of the VIASURE RT-qPCR for N gene.

When testing the six RT-qPCR-positive samples of group 2 (ORF1ab+; Ct > 30/E+ or N+) the RT-LAMP using primer set ORF1ab detected 4/6 samples (66.6%) with shorter (nos. 7, 9, and 10) or similar (no. 8) Tp values than the Ct values obtained by RT-qPCR for both the ORF1ab and the E/N targets. The RT-LAMP using primer set N5 detected 3/6 samples (50%) with much shorter Tp values (nos. 7, 8, 9) than the Ct values obtained for RT-qPCR. The RT-LAMP using primer set N15 amplified 6/6 (100%) samples with relatively long Tp values for samples 8 (Tp = 49), 10 (Tp = 60 min) and 11 (Tp = 45 min) in comparison to the Ct values obtained by RT-qPCR. For samples nos. 9 and 12, the Tp values were similar than RT-qPCR results (Ct = 39 and Ct = 36, respectively). For no. 7, a very short Tp = 13 min was obtained. The RT-LAMP using primer set E only amplified 1/6 samples (16.6%) (sample no. 7), with a Tp = 60 min, a value much longer than the one obtained by RT-qPCR.

The samples nos. 13 and 14 of the group 3 (ORF1ab+; Ct > 30/E−) were amplified by RT-LAMP using the primer set ORF1ab with very similar Tp values than RT-qPCR Ct values. The primer set N5 amplified the sample no. 14 with a very short Tp = 18 min in comparison to Ct = 33 obtained for OFR1ab by RT-qPCR. The primer set N15 amplified the two samples, but not the primer set E.

When testing the six samples included in the group 4 (ORF1ab-/N+), the primer set ORF1ab amplified 3 samples (nos. 15, 16, 18). The primer set N15 detected 5/6 samples (86.3%) that resulted N+ by VIASURE RT-qPCR, nevertheless, the primer set N5 did not amplify any sample. The primer set E, either. In all, considering the few positive results obtained with the primer set E (6/12 confirmed positives (both PCR targets positives); 50%) this RT-LAMP assay was discarded for further testing.

**Figure 5:**
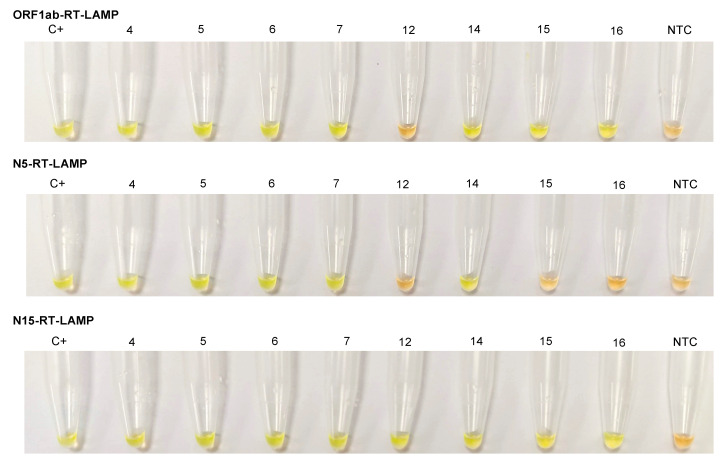
Conventional colorimetric RT-LAMP assays using the primer sets ORF1ab, N5, and N15. Eight RNA isolates from COVID-19 patients (samples nos. 4, 5, 6, 7, 12, 14, 15, and 16) were analyzed by colorimetric RT-LAMP using SYBR Green I fluorescent dye. Green (positive samples), orange (negative samples). C+, RNA-positive control; NTC, non-template control.

To evaluate the conventional colorimetric RT-LAMP assay we selected 8 RNA isolates representing the four groups of samples used in the study: samples nos. 4, 5, 6 (ORF1ab+; Ct ≤ 30/E+ or N+); samples nos. 7, 12 (ORF1ab+; Ct > 30/E+ or N+); sample no. 14 (ORF1ab+/E+), and samples nos. 15, 16 (ORF1ab-/N+). The samples were tested using the primer sets ORF1ab, N5, and N15. The performance of each RT-LAMP assay is shown in Figure 5. Green fluorescence was clearly observed in the successful RT-LAMP reactions, while it remained original orange in the negative reactions. For the selected samples, the color change matched 100% with the results obtained in real-time RT-LAMP assays.

### 3.4. Stability and Functionality Over Time of Dry-RT-LAMP Mixes

**Figure 6:**
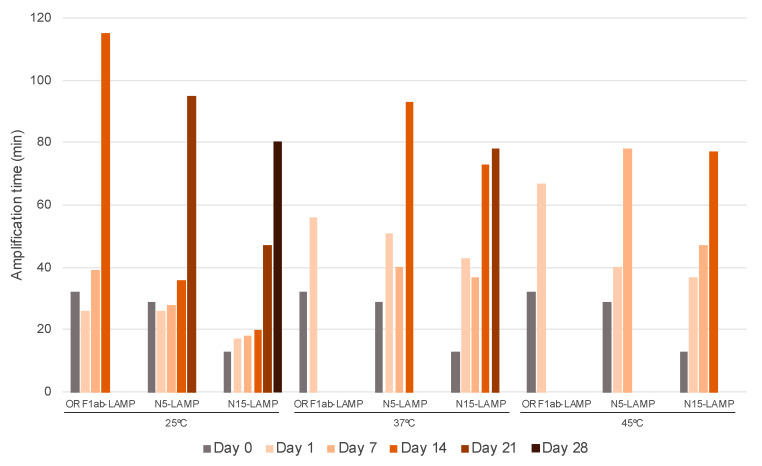
Amplification time of Dry-RT-LAMP assays as a function of storage time and temperature. Amplification times of C+ in RT-LAMP assays performed with dry reagents including primer sets ORF1ab, N5, and N15 tested at 0, 1, 7, 14, 21, and 28-days post-desiccation is shown. The different storage temperature (25 °C, 37 °C and 45 °C) is also indicated.

As the primer sets ORF1ab, N5, and N15 offered the best results in the amplification of the COVID-19 patients’ RNA isolates they were selected for further stabilization assays. The results obtained in Dry-RT-LAMP tests for each primer set are shown in Figure 6. In general, reconstitution of dry reagents worked well, and amplification was obtained for the three primer sets used at RT (25 °C), but a delay in amplification during the reaction for dried mixtures was observed in comparison to fresh mixes. Thus, just after desiccation of reagents (at day 0) an increase in Tp values was noticed for primer sets ORF1ab (Tp = 20.5 min to Tp = 32 min) and N5 (Tp = 19 min to Tp = 29 min). Significantly, no variation of Tp value was registered for primer set N15 after desiccation (Tp = 15 min). Despite the increase in Tp values over time, storage of dry-reagent RT-LAMP assays at RT was found to be functional for 14, 21, and 28 days when using primer sets ORF1ab, N5, and N15, respectively. Remarkably, the Dry-N15-RT-LAMP assay proved to be stable up to 28 days with a very reasonable Tp = 80 min. According to values of the stability times obtained at 37 °C (21 days) and 45 °C (14 days), the *Q*_10_ method predicted up to 66 days and 64 days of shelf-life at room temperature, respectively.

**Figure 7:**
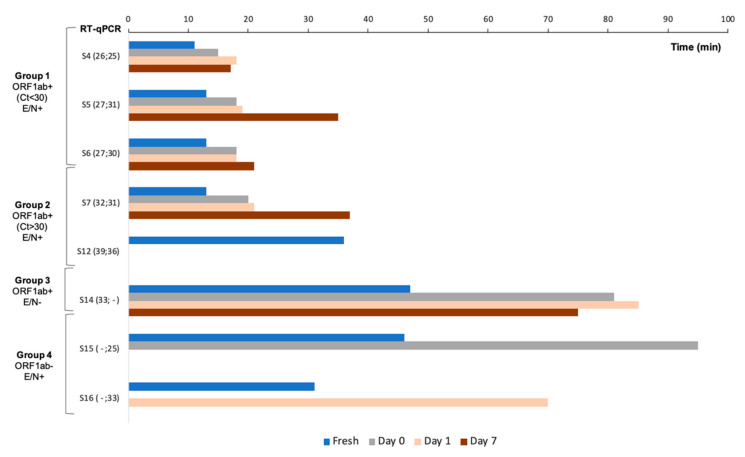
Dry-RT-LAMP assessment using primers set N15 in RNA isolates from COVID-19 patients. Amplification times of samples (S) nos. 4, 5, 6, 7, 12, 14, 15, and 16, performed with dry reagents including primer set N15 at 0, 1, and 7-days post-desiccation are shown. Blue bars (fresh) indicate amplification times obtained in N15-RT-LAMP assays using non-dried reactions (fresh liquid mixes) as reference for comparison. All those reactions were singles.

Dry-N15-RT-LAMP assay was also tested at 0, 1 and 7-days post-desiccation with the same samples used in colorimetric RT-LAMP assays (nos. 4, 5, 6, 7, 12, 14, 15, and 16). Results obtained are shown in Figure 7. As for C+ amplification trials, an increase in the Tp values was observed in comparison to fresh reactions. The only sample with no amplification at any post-desiccation time was the no. 12, which presented the highest Ct values in RT-qPCR for both ORF1b (Ct = 39) and N (Ct = 36). The sample no. 14 (with a high Ct = 33 for ORF1ab and E− by RT-qPCR) was amplified with long Tp values at 0 (Tp = 81 min), 1 (Tp = 85 min) and 7 (Tp = 75 min) days post-desiccation; interestingly, a long Tp = 47 min was also obtained with N15-RT-LAMP fresh mixture. The samples nos. 15 and 16 (with no amplification of ORF1ab and N+ by RT-qPCR) amplified with high Tp values at 0 and 1day post-desiccation, respectively. These samples also presented a relatively long Tp values in fresh N15-RT-LAMP. The samples nos. 4, 5, 6 (group 1; Ct ≤ 30) and 7 (group 2; Ct > 30) were all amplified at 0, 1, and 7-days post-desiccation with very reasonable Tp values in comparison to fresh mixtures.

## 4. Discussion

In this work, five regions from SARS-CoV-2 viral genome were studied, including the open reading frames ORF1a and ORF1b, and genes S, E, and N. For this, eight sequence-specific RT-LAMP primer sets were designed and screened for the detection of the novel coronavirus. Our RT-LAMP reaction was optimized using a well-established RNA-positive control from a COVID-19 patient and primer sets targeting ORF1ab, gene E, and gene N were finally selected. For most RT-LAMP assays detecting SARS-CoV-2, the genes ORF1ab and N have been used as the principal targets for amplification, to ensure specific and sensitive detection [33]. In fact, in our trials, we obtained the best performance in detecting SARS-CoV-2 RNA targeting these two genes, not only in amplification times, but also in analytical sensitivity. First, we established the proper operation of the primer sets designed, specificity, and sensitivity in the amplification of the selected SARS-CoV-2 RNA target sequences. In set-up trials, the RT-LAMP reactions using *Bst* 3.0 DNA polymerase did not work well and, frequently, non-specific amplification was observed. It has been previously described several differences in efficiency of *Bst* 3.0 compared to *Bst* 2.0 WS depending on time and temperature for amplification [38]. As *Bst* 3.0 has an optimal temperature range for amplification between 68–70 °C, it is possible that when testing different temperatures in the optimization of RT-LAMP, a suboptimal annealing of our primer sets occurred, thus probably increasing the irregular and non-specific amplification. In addition, it has been recently described a significantly increased tendency of *Bst* 3.0 to yield false-positive results in comparison to *Bst* 2.0. These unspecific products were characterized by a higher Tm than specific products which also occurred in our *Bst* 3.0 reactions. These false positives have been associated with the interaction of multiple primers and the template switching and terminal transferase activities of the polymerase, combined with a lack of 3′→5′ exonuclease activity [39]. In this sense, although some previously reported RT-LAMP tests for diagnosis of COVID-19 appear to have worked well using *Bst* 3.0 [40,41], different primers sequences targeting gene N were used and then annealing could be probably more effective or be less constrained by primer interaction. Nevertheless, most studies use a combination of a *Bst* 2.0 WS DNA polymerase and *RTx* WS in one-step reaction to amplify gene ORF1ab or gene N [33]. In our trials, optimization of RT-LAMP was finally carried out using the combination of the two enzymes and the best performance resulted for the amplification of regions ORF1ab, E, and N (using both primer sets N5 and N15). The shortest Tp values for RT-LAMP assays were obtained targeting gene N, particularly with primer set N15 (Tp = 15 min). On the other hand, ORF1b was prone to false-positive results so it was removed. Some background signal was detected in the case of E and M primer sets; however; it was not due to DNA amplification, but rather related to the real-time device measurements.

Regarding specificity, RT-LAMP showed to be highly specific for SARS-CoV-2 since no cross-reaction resulted in silico comparisons, although validation with real samples is needed. RNA isolates from other human respiratory viruses were evaluated did not show cross-reaction either. As may occur with any molecular-based test, possible mutations can arise in viral sequences and may affect primer annealing, thus causing a failure in further amplifications. To date, of the mutations reported for SARS-CoV-2 [42], none of them match in the sequences targeted by the primer sets used in RT-LAMP assays. In addition, it seems that mutations (most of them single-nucleotide alterations between viruses from different people) makes SARS-CoV-2 change much more slowly as it spreads [43].

RT-LAMP reactions targeting ORF1ab and N (using sets N5 and N15) demonstrated a limit of detection of 200 copies of SARS-CoV-2 RNA/reaction. The sensitivity of amplification based on gene E detection was proved to be lower (2000 copies/reaction) than ORF1ab or N genes. This might be caused by the slower kinetics of the RT-LAMP reaction targeting gen E, which lacks in loop primers, and therefore does not allow the detection of low concentrations of viral RNA within a reasonable time. Moreover, most of the RT-LAMP assays developed to date do target genes ORF1ab or N [33], and do not gene E, thus suggesting poorer results when using this target. Other published studies have reported sensitivities as low as 2 copies/reaction for both ORF1ab and N genes [28] or 3 copies/reaction for gene ORF1ab [20]. However, those results for analytical sensitivity in detecting SARS-CoV-2 RNA were found using synthesized RNA fragments of genes N and ORF1ab obtained from in vitro transcription instead real RNA isolates from COVID-19 patients. Our results are in line with those larger clinical studies in which RT-LAMP assays present analytical sensitivities around hundreds of copies of SARS-CoV-2 genomic RNA [17,25,44].

A great variability in viral load in COVID-19 patients has been reported, ranging from 641 copies/mL to 1.34 × 10^11^ copies/mL (with a median of 7.99 × 10^4^ in throat samples and 7.52 × 10^5^ in sputum samples) and 1.69 × 10⁵ copies/mL in a nasal swab sample [45]. Other studies testing SARS-CoV-2 positive patients estimated a viral load ranging from 1 copy/μL to 10^8^ copies/μL, with most samples ranging from 10^4^–10^8^ copies/μL [46] or median viral load of 1440 copies/μL in nasopharyngeal swab samples [47]. In addition, a study performed by Yu et al. [48] showed that the viral loads in the early and progressive stages were significantly higher (over 46,000 copies) than in the recovery stage of the disease (over 1200 copies). Despite these variations in viral load of COVID-19 patients, our RT-LAMP assay resulted sensitive enough for detection of SARS-CoV-2 RNA in clinical samples, as it seems to indicate the comparison of Ct values with those Tp values obtained with RT-qPCR. Thus, for samples with a Ct ≤ 30 obtained by RT-qPCR (group 1), we found an excellent sensitivity and specificity values for viral RNA by RT-LAMP assays using primer sets ORF1ab, N5, and N15. The Tp values for the three RT-LAMP assays resulted much shorter than those obtained with RT-qPCR. This fact is even more significant if we take into account that to calculate the Tp of the RT-LAMP assay, both the time dedicated to retro-transcription and amplification are considered, while the Ct of the RT-qPCR does not include the time dedicated to retro-transcription. In general, for clinical samples with Ct > 30 (or with only one RT-qPCR-amplified target; suggestive positives), RT-LAMP assays were initially less sensitive, and amplification was not obtained in all samples. However, the RT-LAMP with the primers set N15, although with high threshold time values, tested positive in all but one sample, probably suggesting a sensitivity significantly lower than 200 copies/reaction for N15-RT-LAMP.

In this work, we have developed three highly efficient RT-LAMP assays for the detection of SARS-CoV-2 RNA. Considering those samples with a RT-qPCR-positive result for two different targets (nos. 1–12; confirmed positives), value of sensitivities resulted in 75% (9/12) for N5-RT-LAMP, 83.3% (10/12) for ORF1ab-RT-LAMP, and 100% (12/12) for N15-RT-LAMP. Furthermore, if only samples nos. 1–10 are considered, with a RT-qPCR Ct ≤ 33 for ORF1ab (equivalent to approximately 10–100 copies), sensitivities of ORF1ab-RT-LAMP and N5-RT-LAMP increase to 100% and 90%, respectively. It should be also noted that Tp values for N5-RT-LAMP were considerably lower than the Ct values for RT-qPCR targeting gene N (nos. 1, 3, 4, 5, 6, 7, and 9), and all positive result could be detected with excellent reaction times under 25 min. All these results suggest that any of the three RT-LAMP assays would be able to detect COVID-19 patients in all disease stages (early, progressive and recovery) according to the currently known data on viral load of SARS-CoV-2 in clinical samples [45,46,47,48]. The significant correlation between RT-qPCR and RT-LAMP threshold times obtained—particularly in theoretically high viral load samples (Ct ≤ 30)—together with the absolute agreement between real-time and conventional colorimetric RT-LAMP assays, increase the confidence in our results. Nevertheless, the increased variability in Tp values that N15-RT-LAMP presented with theoretically low viral load samples (Ct > 30) cannot be disregarded. In this respect, it is important to note that a lack of correlation between speed and sensitivity in isothermal amplification reactions has been previously reported [49] and reactions with higher efficiency can have substantially longer times to be positive, thus contradicting the intuition derived from qPCR reactions. We are aware of the limitations of our study in terms of the sample size and we acknowledge that further studies to examine the reproducibility of N15-RT-LAMP in testing larger sets of clinical samples, both positive and negative, are needed. We also acknowledge the limitations in sensitivity of sets N5 and ORF1ab for the detection of positive samples with low viral load, with RT-qPCR Ct over 33. Those limitations should also be investigated in larger sets of clinical samples.

To develop a RT-LAMP as simple as possible to carry out in any condition for SARS-CoV-2 detection, we tried to keep all necessary components in a non-reactive state using tubes containing dry master mixes coated on the inner walls and caps. In a previous work, we successfully developed a simple desiccation procedure for drying LAMP reagents adapted for conventional and real-time amplifications assays [14]. Now, for COVID-19 RT-LAMP test, that protocol has been modified to achieve better thermal stability of dehydrated RT-LAMP mixes at ambient temperature along time. A Dry-RT-LAMP format can overcomes the requirement of cold storage facilities and temperature-controlled shipping [50,51], allows the omission of adding reagents individually, making the process easier and faster, and avoids possible cross-contamination during multiple pipetting steps in master mix preparation. The new 30 min one-step dry-up protocol was applied for RT-LAMP mixes containing primer sets ORF1ab, N5, and N15, resulting in functional amplifications of the C+ after storage at RT (25 °C) for up 14, 21, and 28 days, respectively. At this moment, we are not aware of the underlying cause of differences in stability when using different primer sets, but it could be possible than the higher efficiency showed by RT-LAMP with the primers set N15 allowed to amplify viral RNA after longer periods of storage. On the other hand, the longer dry components are storage at RT, the longer the reaction incubation time to achieve amplification is needed. An increase in reaction time, as well as a reduction in the amplification level in comparison to fresh liquid mixtures was already described by our group in operation of desiccation LAMP procedure [14]. In any case, very reasonable amplification times of 80 min (for set N15), 95 min (for set N5) and 115 min (for set ORF1ab) were observed before functionality loss after storage for 28, 21, or 14 days, respectively. Additionally, the *Q*_10_ method predicts a shelf-life for Dry-RT-LAMP using primer set N15 of over 64–66 days at 25 °C. Subsequently, the Dry-N15-RT-LAMP format was selected to test those eight samples used in conventional colorimetric RT-LAMP assay at 0, 1, and 7 days post-desiccation. In analysis, an expected increase in the Tp values was observed in comparison to results obtained in N15-RT-LAMP fresh liquid reactions. Thus, those samples with long Tp values when testing in fresh using primer set N15 (presumably with a very low viral load or marginal positives: nos. 12, 14, 15, and 16) did not work very well at post-desiccation times, resulting in no amplification or in amplification with Tp values much longer. By contrast, those samples with short Tp values in fresh testing (presumably with medium/high viral load: nos. 4, 5, 6 and 7), despite increase slightly in reaction time, were consistently detected over post-desiccation time.

In summary, we have developed a novel, rapid, specific and sensitive RT-LAMP test for SARS-CoV-2 RNA detection in clinical samples by targeting gene N with a specific-sequence primer set N15. Our RT-LAMP assay can be simply performed both as a single-tube isothermal colorimetric method without any expensive equipment requirement and in a real-time platform. The results can be detected as soon as 9 min after the reaction starts and obtain close to 100% sensitivity within 60 min. Moreover, the procedure is easily adaptable to a dry format that could be stored and delivered at room temperature. At this moment, maintaining the functionality for at least 2 months at RT, would allow us to prepare and distribute a set of dried RT-LAMP master mixes to be used within a few weeks in settings where detection of SARS-CoV-2 is required at the point of collection, such as schools, nursing homes, or rural medical centers. This feature, which can be achieved by a simple and fast process in comparison to other available options, mainly lyophilization, could represent a great contribution to fast molecular SARS-CoV-2 diagnostic tools. Additionally, the affordability of the test is apparent, as the price per reaction in this study was 1.76 € for fresh mixes when using SYBR Green I and 1.69 € when using Eva Green 20x. The desiccation process only added 0.02 € per reaction. However, it is important to highlight that RNA purification would add over 3.50 € per sample depending on the commercial extraction kit used. Thus, the price per reaction is significantly cheaper than a standard RT-qPCR test which is approximately 7–10 € without considering previous RNA purification. Notwithstanding its limitations, the possibility of avoid the RNA extraction, or the combination of this RT-LAMP with some rapid RNA purification methods already described [22,34,52], could allow easy testing in situations of rapid diagnostic demand and in low-resource settings and areas of difficult access, where the limited testing capacity is one of the main challenges in the COVID-19 response [53], reducing considerably its price too. More work on this with the aim to improve and achieve a point-of-care (POC) molecular diagnosis of COVID-19 will be performed in the future.

## Figures and Tables

**Table 1:** Primer sets used in this study for the detection of SARS-CoV-2 through reverse-transcription loop-mediated isothermal amplification (RT-LAMP).

Set	Primer ^1^	Sequence 5′→3′	Length (nt)	Gene Position
ORF1ab ^2^	F3	TGCTTCAGTCAGCTGATG	18	13,434–13,636
	B3	TTAAATTGTCATCTTCGTCCTT	22	
	FIP	CAGTACTAGTGCCTGTGCCGCACAATCGTTTTTAAACGGGT	41	
	BIP	TCGTATACAGGGCTTTTGACATCTATCTTGGAAGCGACAACAA	43	
	LF	CTGCACTTACACCGCAA	17	
	LB	GTAGCTGGTTTTGCTAAATTCC	22	
ORF1b ^3^	F3	CACAGACTTTGTGAATGAGTT	21	15,654–15,896
	B3	GTCAGTCTCAGTCCAACAT	19	
	FIP	CTATTGAAACACACAACAGCATCGCATATTTGCGTAAACATTTCTCA	47	
	BIP	TATGCATCTCAAGGTCTAGTGGCTATGCTTCAGACATAAAAACATTG	47	
S447 ^3^	F3	GTTTCTGCCTTTCCAACAA	19	22,985–23,415
	B3	AACAGGGACTTCTGTGCA	18	
	FIP	TCAAGAATCTCAAGTGTCTGTGGTGGCAGAGACATTGCTGA	41	
	BIP	ACCATGTTCTTTTGGTGGTGTCAACATCCTGATAAAGAACAGC	43	
	LF	TCACGGACAGCATCAGTAGTG	21	
	LB	CAGGAACAAATACTTCTAACCAGGT	25	
S555 ^3^	F3	CTATGCAAATGGCTTATAGGTT	22	24,182–24,736
	B3	AGTTGTTTAACAAGCGTGTT	20	
	FIP	GCACTATTAAATTGGTTGGCAATCATAATGGTATTGGAGTTACACAGA	48	
	BIP	ATTGGCAAAATTCAAGACTCACTTTTGTGCATTTTGGTTGACC	43	
E ^4^	F3	TCATTCGTTTCGGAAGAGA	19	26,245–26,472
	B3	AGGAACTCTAGAAGAATTCAGAT	23	
	FIP	TGTAACTAGCAAGAATACCACGAAACAGGTACGTTAATAGTTAATAGCG	49	
	BIP	GCTTCGATTGTGTGCGTACTCGAGAGTAAACGTAAAAAGAAGG	43	
M ^4^	F3	GTTTCCTATTCCTTACATGGATT	23	26,597–26,801
	B3	AGCCACATCAAGCCTACA	18	
	FIP	CCATAACAGCCAGAGGAAAATTAACCTTCTACAATTTGCCTATGCC	46	
	BIP	AACTTTAGCTTGTTTTGTGCTTGCACAAGCCATTGCGATAGC	42	
N5 ^4^	F3	CCAGAATGGAGAACGCAGTG	20	28,355–28,570
	B3	CCGTCACCACCACGAATT	18	
	FIP	AGCGGTGAACCAAGACGCAGGGCGCGATCAAAACAACG	38	
	BIP	AATTCCCTCGAGGACAAGGCGAGCTCTTCGGTAGTAGCCAA	41	
	LF	ATTATTGGGTAAACCTTGGGGC	22	
	LB	ATTAACACCAATAGCAGTCCAGATG	25	
N15 ^4^	F3	AGATCACATTGGCACCCG	18	28,703–28,915
	B3	CCATTGCCAGCCATTCTAGC	20	
	FIP	TGCTCCCTTCTGCGTAGAAGCCAATGCTGCAATCGTGCTAC	41	
	BIP	GGCGGCAGTCAAGCCTCTTCCCTACTGCTGCCTGGAGTT	39	
	LF	GCAATGTTGTTCCTTGAGGAAGTT	24	
	LB	CCTCATCACGTAGTCGCAACAG	22	

F3, forward primer; B3, backward primer; BIP, backward inner primer; FIP, forward inner primer; LB, loop backward; LF, loop forward; ^1^ Primer concentrations remain unchanged for all sets: 1.6 μM FIP/BIP, 0.2 μM F3/B3, 0.4 μM LF/LB; ^2^ Primer set previously described by El-Tholoh et al. [24]; ^3^ Primer sets (original design) based upon sequences used for RT-qPCR detection of SARS-CoV-2 by Wu et al. [35]; ^4^ Primer sets (original design).

**Table 2:** Comparison of the results of cycle threshold (Ct) values obtained by RT-qPCR and time to positivity (Tp) values obtained by RT-LAMP assays using primer sets ORF1ab, N5, N15, and E in testing 20 RNA isolates from COVID-19 patients. Groups of samples, commercial Real-Time PCR Detection Kits targeting ORF1ab and E/N used in RT-qPCR tests, and primer sets evaluated for RT-LAMP assays are indicated. Ct and Tp values are indicated in minutes.

		RT-qPCR Ct Values			Real-Time RT-LAMP Tp Values			
Groups	No. Sample	Commercial Kit ^1^	ORF1ab	E/N	ORF1ab	N5	N15	E
Group 1 ORF1ab+ (Ct < 30) E/N+	1	VIASURE	22	27	15	14	9	27
	2	COBAS	25	26	18	16	13	72
	3	VIASURE	25	29	15	15	19	43
	4	VIASURE	26	25	17	16	11	28
	5	VIASURE	27	31	17	18	13	-
	6	VIASURE	27	30	19	18	13	35
Group 2 ORF1ab+ (Ct > 30) E/N+	7	VIASURE	32	31	20	22	13	60
	8	COBAS	32	35	35	23	49	-
	9	VIASURE	33	39	23	20	39	-
	10	COBAS	33	36	31	-	60	-
	11	COBAS	34	36	-	-	45	-
	12	VIASURE	39	36	-	-	36	-
Group 3 ORF1ab+ E−	13	COBAS	36	-	39	-	29	-
	14	COBAS	33	-	30	18	47	-
Group 4 ORF1ab- N+	15	VIASURE	-	25	34	-	46	-
	16	VIASURE	-	38	28	-	31	-
	17	VIASURE	-	38	-	-	-	-
	18	VIASURE	-	40	43	-	60	-
	19	VIASURE	-	41	-	-	58	-
	20	VIASURE	-	41	-	-	30	-

^1^ A different commercial kit for RNA extraction was used to perform RT-qPCR using the commercial Real-Time PCR Detection Kits. NUCLISENS EASYMAG, BIOMÉRIEUX, France, for VIASURE SARS-CoV-2 Real-Time PCR Detection Kit, CERTEST BIOTECH, Spain; an integrated system in an automated platform for COBAS 6800, ROCHE, Switzerland, following manufactures’ instructions.
